# Dynamics of two methanogenic microbiomes incubated in polycyclic aromatic hydrocarbons, naphthenic acids, and oil field produced water

**DOI:** 10.1186/s13068-017-0812-2

**Published:** 2017-05-11

**Authors:** Bonahis J. Oko, Yu Tao, David C. Stuckey

**Affiliations:** Department of Chemical Engineering, Imperial College London, Exhibition Road, South Kensington, London, SW7 2AZ UK

**Keywords:** Microbial community, Produced water, Naphthenic acids, Polycyclic aromatic hydrocarbons, Bioaugmentation

## Abstract

**Background:**

Oil field produced water (OFPW) is widely produced in large volumes around the world. Transforming the organic matter in OFPW into bioenergy, such as biomethane, is one promising way to sustainability. However, OFPW is difficult to biologically degrade because it contains complex compounds such as naphthenic acids (NAs), or polycyclic aromatic hydrocarbons (PAHs). Although active microbial communities have been found in many oil reservoirs, little is known about how an exotic microbiome, e.g. the one which originates from municipal wastewater treatment plants, would evolve when incubated with OFPW.

**Results:**

In this study, we harvested methanogenic biomass from two sources: a full-scale anaerobic digester (AD) treating oil and gas processing wastewater (named O&G sludge), and from a full-scale AD reactor treating multiple fractions of municipal solid wastes (named MS, short for mixed sludge). Both were incubated in replicate microcosms fed with PAHs, NAs, or OFPW. The results showed that the PAHs, NAs, and OFPW feeds could rapidly alter the methanogenic microbiomes, even after 14 days, while the O&G sludge adapted faster than the mixed sludge in all the incubations. Two rarely reported microorganisms, a hydrogenotrophic methanogen *Candidatus methanoregula* and a saccharolytic fermenter *Kosmotoga*, were found to be prevalent in the PAHs and OFPW microcosms, and are likely to play an important role in the syntrophic degradation of PAHs and OFPW, cooperating with methanogens such as *Methanoregula, Methanosarcina, or Methanobacterium*.

**Conclusions:**

The dominant phyla varied in certain patterns during the incubations, depending on the biomass source, feed type, and variation in nutrients. The sludge that originated from the oil and gas processing wastewater treatment (O&G) reactor adapted faster than the one from municipal solid waste reactors, almost certainly because the O&G biomass had been “pre-selected” by the environment. This study reveals the importance of biomass selection for other crude oil-waste-related bioengineering studies, such as bioaugmentation and bioremediation.

**Electronic supplementary material:**

The online version of this article (doi:10.1186/s13068-017-0812-2) contains supplementary material, which is available to authorized users.

## Background

Recalcitrant compounds possess the ability to remain and persist in a particular environment, although some can be degraded by microorganisms at a very low efficiency [1, 2]. There are two major categories of recalcitrant compounds, i.e. natural compounds (e.g. humic substances, lignin, and halogenated compounds) and anthropogenic (man-made) ones. The latter are often more refractory due to a lack of necessary enzymes that are excreted by naturally present microorganisms, or these enzymes are blocked [3]. A typical industry-oriented recalcitrant waste is oil field produced water (OFPW), and this contains complex recalcitrant compounds, including naphthenic acids (NAs) [4] and polycyclic aromatic hydrocarbons (PAHs) [5]. Hence, it is difficult to treat using conventional aerobic-activated sludge.

Interestingly, a variety of microorganisms have been identified in oil fields [3, 6, 7]. Among these diverse communities, anaerobic microorganisms, e.g. methanogenic Archaea, play a vital role in degrading recalcitrant compounds [8–10]. Hence, it is eminently reasonable to attempt to engineer an anaerobic digestion process in bioreactors for recovering bioenergy (biogas) from OFPW. However, it is difficult to achieve satisfactory performance in these systems due to the lack of sufficient numbers of specialised microorganisms that contain key metabolic routes to mineralise the target contaminants [11].

Bioaugmentation, one way to bio-catalyse a degradation process, offers a novel pathway to degrade recalcitrant compounds. Since it utilises the microbial consortia that are assembled for a specific physicochemical process, bioaugmentation should be more efficient in treating recalcitrant compounds than the use of an undefined inocula [12]. The microorganisms that can be used in bioaugmentation should meet three criteria: they should be (1) catabolically able to degrade the contaminant, even in the presence of other potentially inhibitory pollutants, (2) competitive after their introduction to a treatment system, and (3) compatible with the indigenous communities present [13]. Therefore, a candidate microbial assembly should be carefully selected to achieve a successful bioaugmentation. However, a simple and effective strategy for selecting the optimal microbial community is still poorly understood. We argue that an important step prior to achieving such a strategy is to understand how a methanogenic microbiome would change dynamically (over time) in the presence of OFPW or OFPW-related compounds. For this reason, our aim in this paper was to elucidate the dynamic patterns of methanogenic microbiomes when introducing PAHs, NAs, and OFPW to the cultures under anaerobic conditions. We used a batch incubation method accompanied by the next-generation sequencing tools to select and identify microbial communities that contained desirable catabolic traits to degrade PAHs, NAs, and OFPW. This study is expected to help in the development of a strategy for selecting optimal microbial cultures for bioaugmenting the treatment of OFPW.

## Results

### Microbiome dynamics in the PAH consortia

**Fig. 1:**
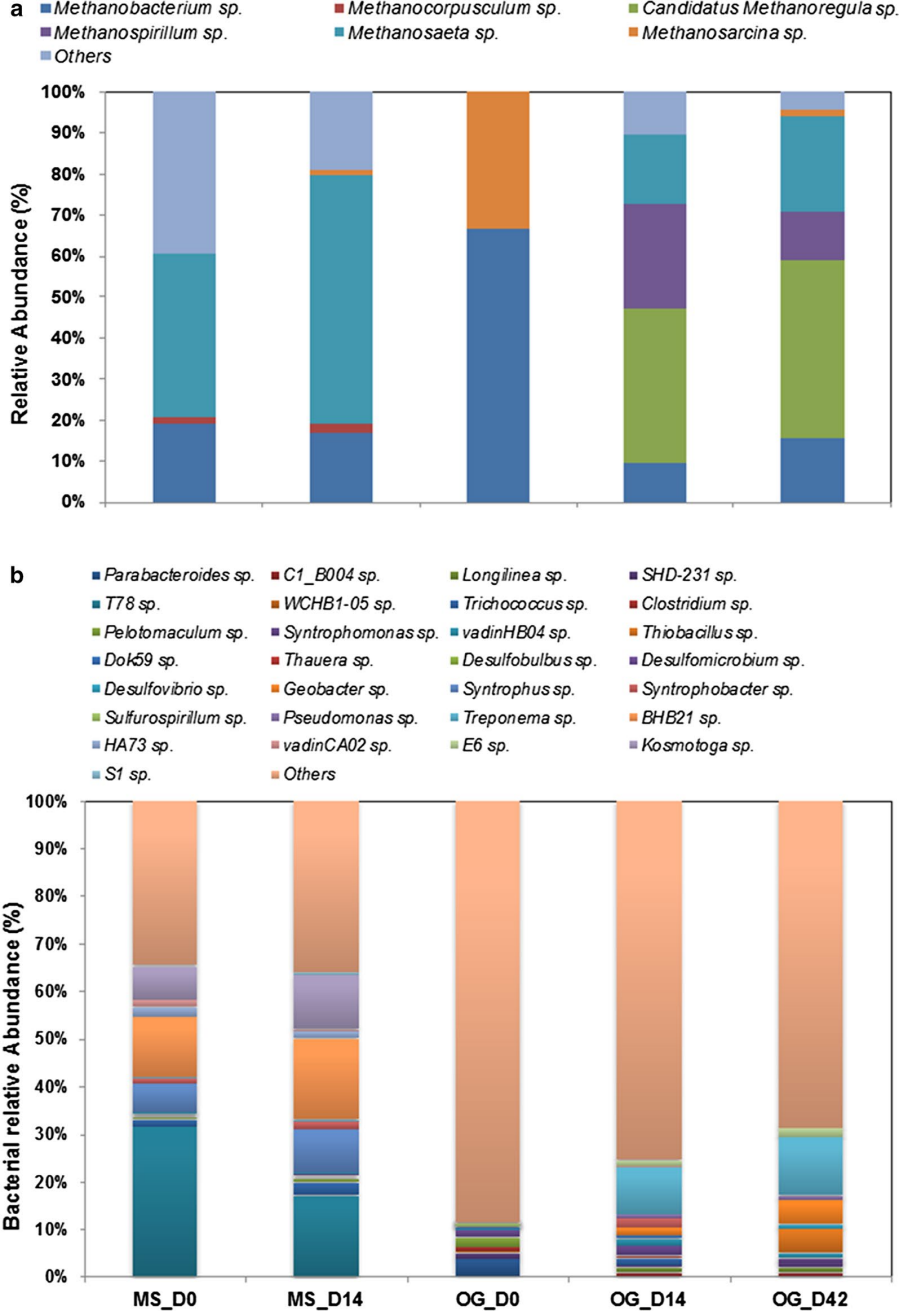
The relative abundance of each key genus in the methanogenic community (**a**) and the bacterial microbiome (**b**) in the PAHs enrichment cultures

There were 14 and 17 phyla identified in the mixed sludge and O&G sludge (both were inocula), respectively (Additional file 1: Figure S1). Euryarchaeota was the dominant archaeal phylum in the two inocula, and its relative abundance increased from 5.8 to 8.9% within the initial 14 days of incubation in the MS group and increased from 0.6 to 10.2% within 14 days in the O&G sludge group. The dominant bacterial phyla in the MS community were Chloroflexi (18.1%) and Spirochaetes (16.5%) after a 14-day incubation, while the O&G sludge community was dominated by bacteroides whose abundance decreased slightly from 43% after 14 days to ~40% by the end of incubation on day 42. For the methanogenic community, the acetoclastic methanogen *Methanosaeta* had a relative abundance of 40% in the MS community on day 0 (Fig. 1a), followed by the hydrogenotrophic methanogen *Methanobacterium* (~20%). After the first 14-days of incubation, *Methanosaeta* accounted for >60% of the archaeal gene population, while the relative abundance of *Methanobacterium* decreased to ~18%. The O&G sludge contained a different archaeal community; *Methanobacterium* was the most dominant genus (67%) on day 0, followed by the versatile methanogenic genus *Methanosarcina* (33%). However, the previously low-abundance *Methanoregula* became the most dominant methanogenic genus (43%) after the 42-day incubation, followed by *Methanosaeta* (24%) and *Methanobacterium* (15%).

One of the dominant bacterial genera that were identified in the mixed sludge was T78 (Fig. 1b). This is a Chloroflexi green non-sulphur bacterium that has been reported to dominate the methane-rich Santa Barbara basin sediments underlying sulphate–methane transition zones [14]. Its relative abundance decreased by 15% in two weeks from 31% (day 0) to 17% (day 14) in the MS community. BHB21 was another dominant genus with a relative abundance of 13 and 17% on day 0 and 14, respectively. Besides T78 and BHB21, the relative abundance of the genus *Kosmotoga* also increased from 11% (day 0) to 18% (day 14). Within the O&G sludge community, the dominant bacterial genus was *Treponema,* and its abundance increased from 1% on day 0 to 10 and 12% on day 14 and 42, respectively. *Thiobacillus* and *Geobacter* were also enriched during the incubation, with their abundance increasing to 6% by day 42. *Geobacter* is a strictly anaerobic bacterium that grows with Fe^3+^ acting as the sole electron acceptor, and is one of the species known to use toluene as an electron donor [15].

**Fig. 2:**
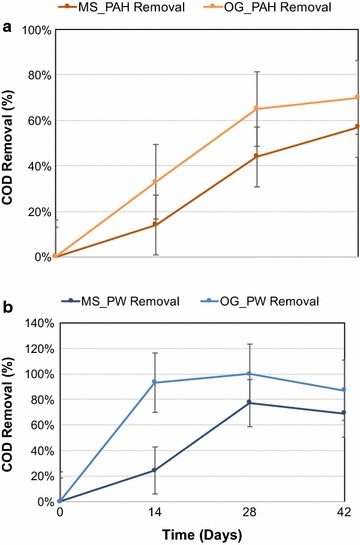
COD removal efficiencies in the PAH (**a**) and OFPW (**b**) enrichment cultures

The changes in the *α*-diversity of the mixed sludge and the O&G sludge communities were characterised by a richness and an evenness index. In general, the bacterial community had a higher richness and evenness than that of the archaeal community for both groups. The evenness (Table 1) appeared to be influenced by the presence of the contaminant (PAHs) rather than the inoculum source, while the richness appeared to be influenced more by the inoculum source than by PAHs. A higher value for the richness was observed with the O&G sludge communities, and it is notable that the COD removal efficiency of the O&G groups (70% on day 42) was also higher than the MS groups (57% on day 42) (Fig. 2a).

**Table 1:** *α*-diversity indices of the microbial communities in the incubations with PAHs, NAs, and OFPW

Sample	OSN	Chao1	Shannon	InvSimpson
PAH cultures				
MS_D0	1197	1735	4.2	21.3
MS_D14	1115	1664	4.3	25.1
OG_D0	1021	1446	3.6	8.9
OG_D42	1579	2321	4.6	23.5
NA cultures				
OG_D0	958	1519	3.5	12.4
MS_D0	1010	1565	3.1	8.6
50% BMP (150 mg L^−1^)_D14	622	1096	1.9	3.1
50% BMP (500 mg L^−1^)_D14	568	924	1.7	2.5
200% BMP (500 mg L^−1^)_D14	534	1001	1.9	3.0
OFPW cultures				
MS_0	1422	2069	4.4	27.3
MS_14	1312	1959	4.6	33.3
MS_28	1162	1746	4.7	40.0
MS_42	1007	1680	4.5	30.5
OG_0	1612	2170	4.4	25.6
OG_14	1137	1647	3.3	5.4
OG_28	1079	1553	4.1	16.8
OG_42	1545	2136	4.6	33.0

**Fig. 3:**
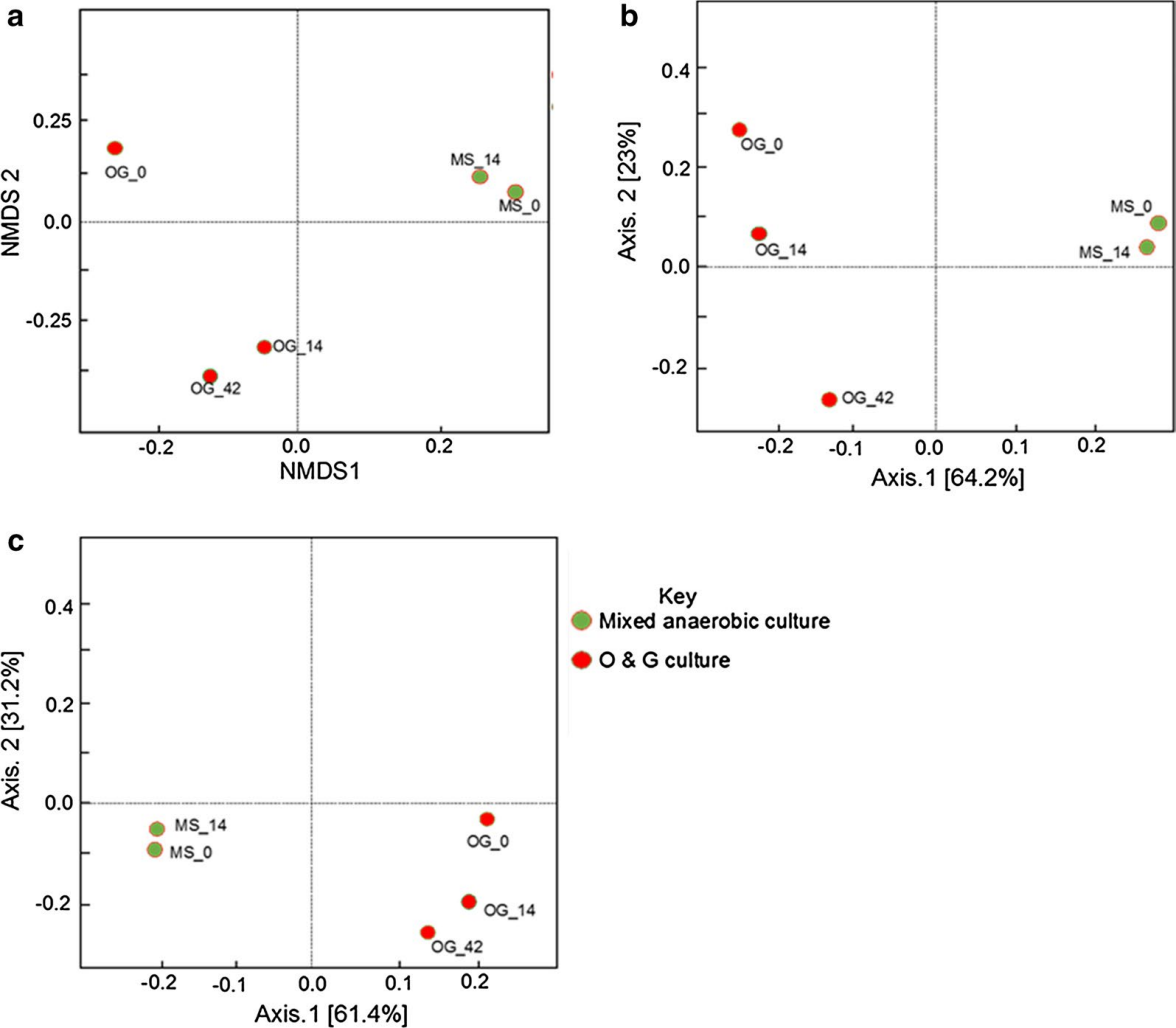
Ordination of the PAHs enrichment sample-based OTUs. **a** Non-metric multidimensional scaling (NMDS) based on Bray-Curtis similarity matrices of OTUs. **b** Principal coordinates analysis (PCoA) based on weighted Unifrac distance matrices of OTUs. **c** PCoA based on unweighted Unifrac distance matrices of OTUs

The NMDS (Bray-Curtis) measure of dissimilarity (Fig. 3a) showed obvious changes in the O&G community from day 0 to 42, which agrees with the *α*-diversity index of observed species number (OSN) and Chao1. Unlike the O&G community, the MS community was more stable, which also agrees with the *α*-diversity results (Table 1), where the values were relatively constant with the evolution of time. The results remained consistent with PCoA weighted (Fig. 3b) and unweighted UniFrac plots analysis (Fig. 3c), which reveal a clear evolution of the microbial community in the O&G group, but only a slight change in the mixed group.

### Microbiome dynamics in the NA consortia

**Fig. 4:**
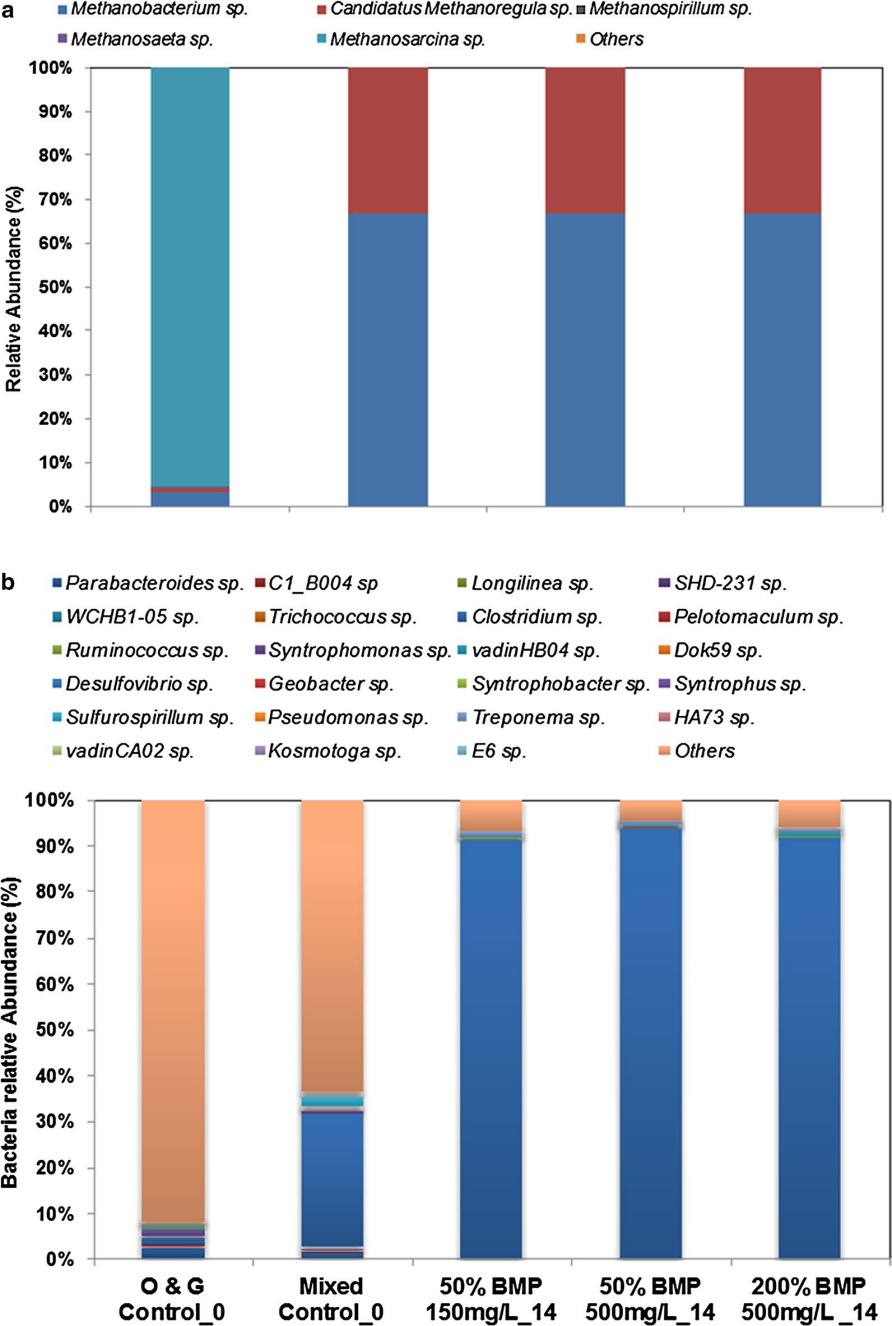
The relative abundance of each key genus in the methanogenic community (**a**) and bacterial microbiome (**b**) in the NA enrichment cultures

Firmicutes were the dominant bacterial phylum (Additional file 1: Figure S2), with a clear increase in its relative abundance from 3% (day 0) to 54% with (50% BMP + 150 mg L^−1^ NAs) and 93% (50% BMP + 500 mg L^−1^ NAs). The dominant archaeal genus (Fig. 4a) identified in the day-0 communities of both mixed and O&G sludge was *Methanosarcina,* with a relative abundance >95% in each sludge. However, a clear evolution in the microbial community occurred by day 14, with *Methanobacterium* occupying the sole dominant role, and whose relative abundance increased to 65 from 5% (day 0). The second dominant genus was *Methanoregula* whose relative abundance increased from 2% (day 0) to 30% (day 14). The dominant bacterial genus was identified as *Clostridium*, whose relative abundance increased from 26% (day 0) to 90% (day 14) in all incubations (Fig. 4b).

**Fig. 5:**
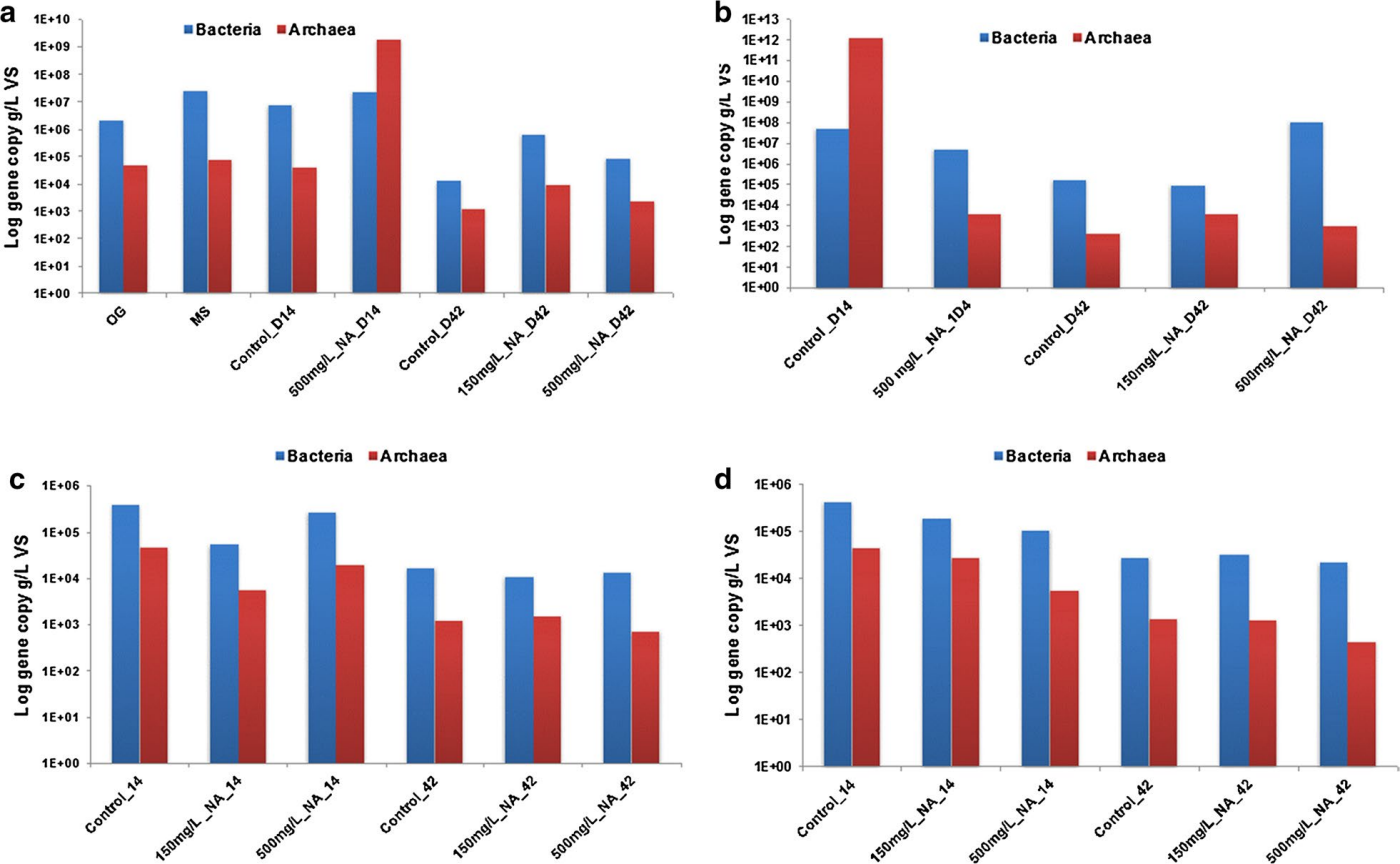
The bacterial and archaeal 16S gene copy concentrations in the NAs consortia revealed by qPCR analysis. **a** 50% BMP media; **b** −100% BMP media; **c** 200% BMP media; **d** 400% BMP media

Analysis of the microbial 16S gene copy concentration in NA enrichment cultures at varying BMP media concentrations (Fig. 5) shows that with all BMP concentrations the number of both the bacterial and archaeal 16S genes decreased with an increase in the concentration of NA from 150 to 500 mg L^−1^, and with an increase in BMP media concentration exceeding 100%. For example, for the group incubated in 50% BMP + 150 mg L^−1^ NAs, the bacterial 16S gene copy concentration was 10^5^ gene copies per gramme sludge, while for the 50% BMP + 500 mg L^−1^ NAs group, the value was almost one order of magnitude lower.

The mixed and O&G sludge communities degrading NAs had been “primed” by degrading NAs prior to being utilised for enrichment cultures. Thus on day 0, both mixed and O&G sludge shared a similar level of richness and evenness (Table 1). However, the *α*-diversity indices decreased over time, indicating a likely toxicity of NAs to the microbial communities.

**Fig. 6:**
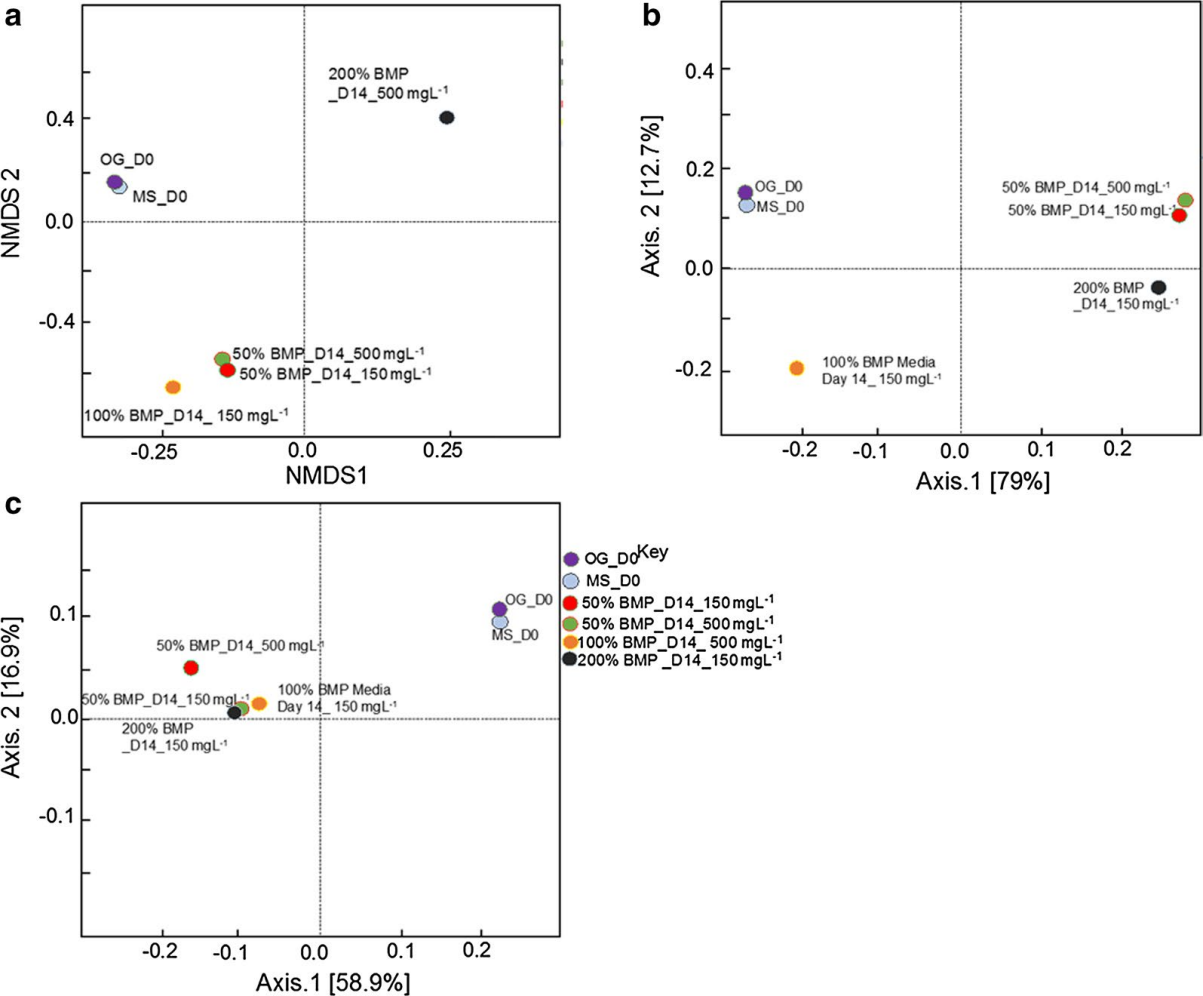
Ordination of the PAH enrichment sample-based OTUs. **a** NMDS based on Bray-Curtis similarity matrices of OTUs. **b** PCoA based on weighted Unifrac distance matrices of OTUs. **c** PCoA based on unweighted Unifrac distance matrices of OTUs

Interestingly, the PCoA weighted UniFrac figure (Fig. 6a) shows that the O&G and mixed sludge community on day 0 showed similar phylogenetic branches (because of priming) (Fig. 6), and this agrees with the *α*-diversity analysis (Table 1). The rare phylogenetic branches identified with PCoA unweighted UniFrac (Fig. 6c) indicated that branches were shared based on contaminant degraded. This hypothesis would agree with the results from the PAHs incubations with O&G biomass (Table 1).

### Microbiome dynamics in the OFPW consortia

In the MS community, 14 different phyla (Additional file 1: Figure S3) were observed on day 42, and the dominant phyla were Chloroflexi, Proteobacteria, Thermotogae, and Synergistetes, with a relative abundance of 30, 18, 16, and 13%, respectively. Within the O&G group, there were 16 different phyla identified, with Bacteroidetes and Spirochaetes co-dominating the community. Their relative abundances increased from 20% (days 0) to 30% (day 42), and from 6.7% (day 0) to 19% (day 42), respectively. In contrast, the phylum Firmicutes decreased from 63% (day 0) to 9.6% (day 42).

**Fig. 7:**
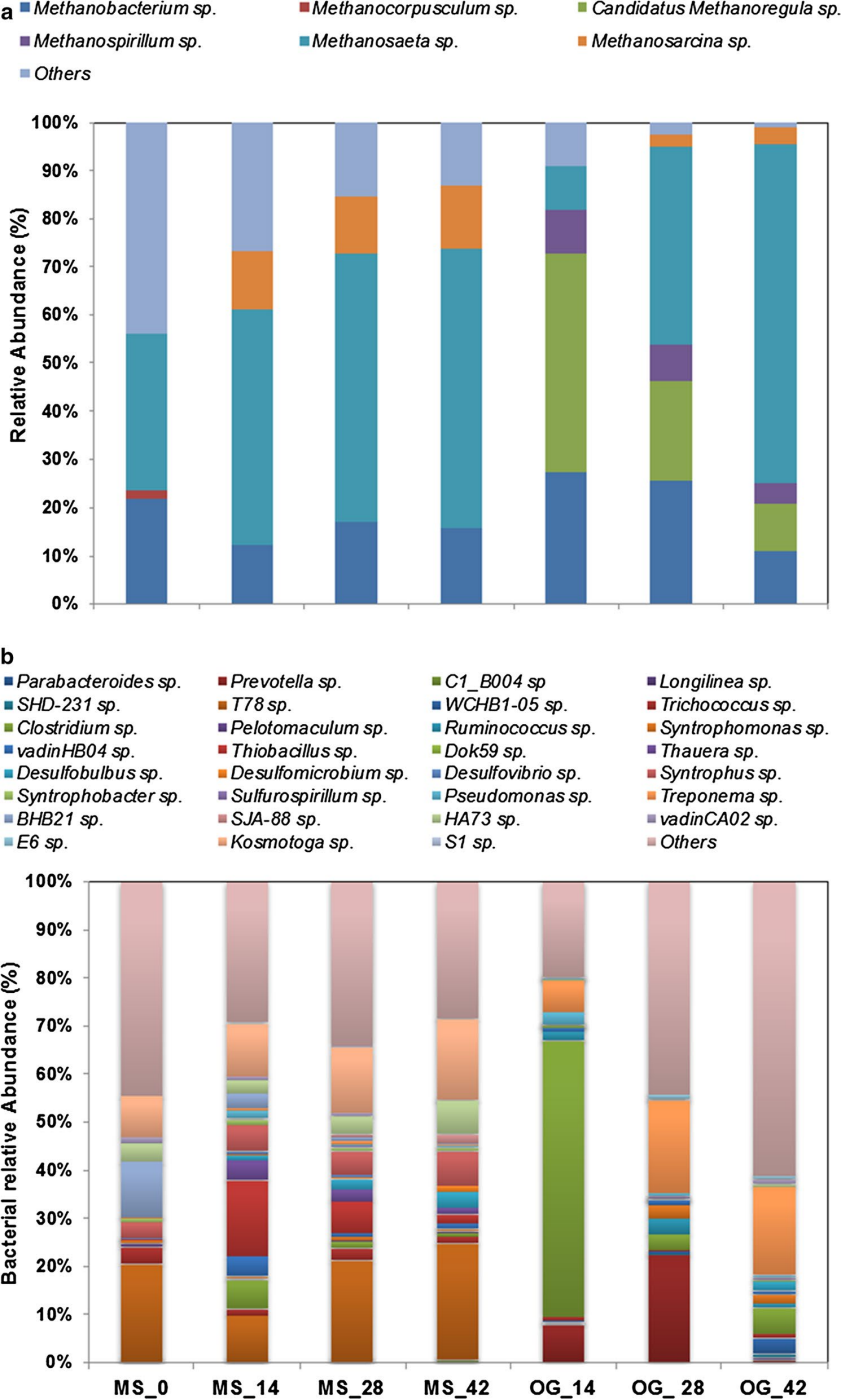
The relative abundance of each key genus in the methanogenic community (**a**) and bacterial microbiome (**b**) genera in the OFPW enrichment cultures

A clear evolution of the archaeal community can be observed in both groups (Fig. 7). Within the MS group, *Methanosaeta* was the dominant genus, with its relative abundance increasing from 30% (day 0) to 60% (day 42). *Methanobacterium* was the second dominant genus, although its relative abundance decreased from 22% on day 0 to 17% on day 42. *Methanosarcina* remained relatively constant around 12% during the incubation. For the O&G group, *Methanosaeta* was the most dominant genus with its relative abundance increasing from 10% (day 0) to 70% (day 42). The relative abundance of *Methanoregula* decreased from 45 to 10%, and that of *Methanobacterium* decreased from 28 to 10%. Within the MS group, *T78* remained relatively constant in abundance and accounted for a maximum of 24%, increasing only 5% during the 42-day incubation. *Kosmotoga* was the next dominant genus, with its relative abundance increasing from 10 to 17% by day 42. *Desulfobulbus* and alkane-oxidising genus *Syntrophus* both accounted for about 7% in the bacterial community. Within the O&G group, *Clostridium* dominated the community with a relative abundance of 57% on day 14, but gradually decreased to 7% by day 42. The relative abundance of *Prevotella* accounted for 22% on day 28, which was much higher than the earlier number of 10% on day 14. However, its dominant role was replaced by *Treponema* by day 42, which increased in abundance from 6.4 to 20%.

**Fig. 8:**
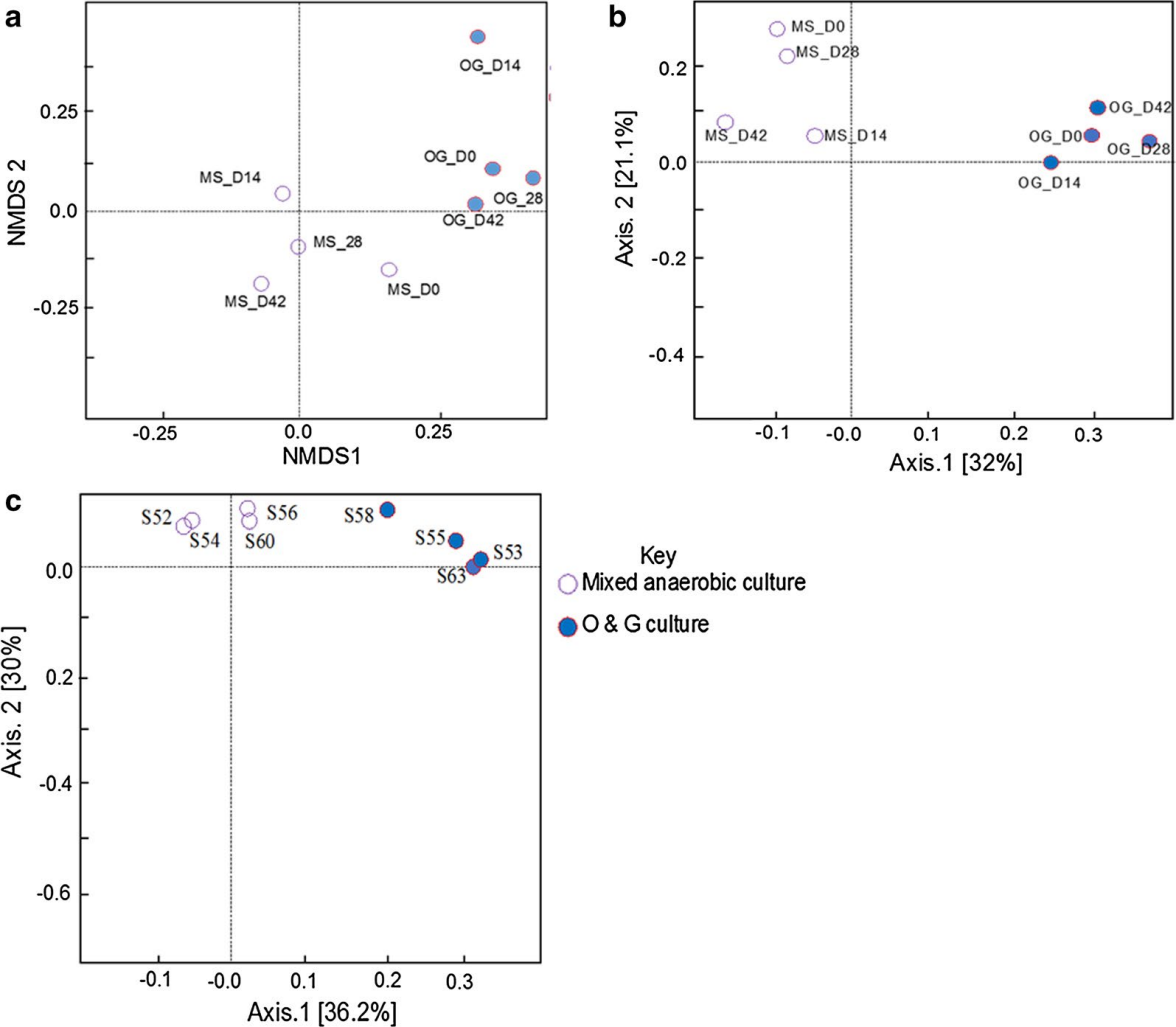
Ordination of the OFPW enrichment sample-based OTUs. **a** NMDS based on Bray-Curtis similarity matrices of OTUs. **b** PCoA based on weighted Unifrac distance matrices of OTUs. **c** PCoA based on unweighted Unifrac distance matrices of OTUs

The *α*-diversity results for the OFPW consortia (Table 1) showed that the richness decreased with time within the MS group, while the evenness increased from 28 to a maximum of 40 within 28 days. Within the O&G community, both species richness and evenness increased with time. The *β*-diversity results (Fig. 8a–c) showed that community profiles were relatively similar between the mixed assays, but the O&G communities appear quite dynamic with the evolution of time. Not surprisingly, we also observed that the COD removal of the O&G group (87% on day 42) was higher than that of the MS group (69% on day 42) (Fig. 2b) since they were better adapted to the degradation of these recalcitrants.

## Discussion

### The roles of key microbial guilds

Some of the dominant genera identified in our experiments have also been identified in previous work. For example, Firmicutes and Proteobacteria were both identified as dominant phyla in petroleum samples from the Brazilian oil fields [7]. Investigation of the microbial community structures in the OFPW obtained from a high-temperature oil formation in the North Sea also confirmed the dominance of Firmicutes, Bacteroidetes, Spirochaetes, Thermotogales, and Proteobacteria in addition to several archaea [16]. In other work, Zhao et al. [17] confirmed that Proteobacteria, Chloroflexi, and Thermotogae were prevalent in water-flooded oil reservoirs in China, and all these three phyla were identified as dominant in our MS consortia after 42-d incubation with OFPW (Additional file 1: Figure S3).

*Clostridium* accounted for almost 90% of the bacterial community in the NA incubations (Fig. 4b), and it is likely they contributed to the degradation of NA compounds and other hydrocarbons [18, 19]. It was dominant in the NA consortia, with the abundance varying from 25% on day 0 to a maximum of >90% in all NA communities (Fig. 4b). *Clostridium* has frequently been reported as an abundant genus in AD microbial communities under either gentle [20], or near-extreme [21] conditions, and it has been suggested that it produces gases, acids, alcohols, and surfactants in the enhanced microbial recovery of heavy oil [22]. It has also been identified as a dominant genus in anaerobic NA-degrading cultures, possibly playing a syntrophic role with methanogenic species in the degradation of NAs [19].

It is interesting that *Methanoregula* was obviously enriched in both the PAHs and NAs media, and even dominated the archaeal community (43% in the PAHs consortia and >30% in NAs). It is a hydrogenotrophic methanogen which can maintain very low H_2_ pressures under propionate-oxidising environments, and allows syntrophic bacteria to produce acetate for *Methanosarcina* in some anoxic environments, such as peatlands [23]. *Methanobacterium and Methanosaeta* were the dominant methanogens in the PAH and OFPW incubations, with obvious variations throughout the incubations (Figs. 1b, 7b). *Methanobacterium* are hydrogenotrophic methanogens [24], while *Methanosaeta*, on the other hand, are typical acetoclastic methanogens that prevail in many anaerobic biogas systems with low COD concentrations in the effluent [25]. *Methanosarcina* was also observed in our incubations, and their versatile metabolism (utilising both acetate and H_2_/CO_2_) enables them to prevail in many other anaerobic habitats, especially under harsh environments [26, 27].

### Rarely identified genus in AD found boosted with bioaugmentation

It was interesting to identify some genera prevalent in our incubations that are rare in AD systems. For example, the relative abundance of genus *Kosmotoga* increased in 14 days in both the PAHs (by 40%) and OFPW (by 22% within 14 days and 51% within 42 days) incubations (Figs. 1, 7). *Kosmotoga* belongs to the order Thermotogales, and its first strain was isolated from an oil production fluid in the North Sea [28]. Although most of the reported *Kosmotoga* members are thermophilic bacteria, some can still grow well at mesophilic temperatures such as 37 °C [27, 29] or even lower [30] despite low abundances. It has been identified with extremely high abundance in a seawater-containing anaerobic digester located in a Hong Kong wastewater treatment plant [31]. Although the physiology and metabolism of *Kosmotoga*, especially in anaerobic digestion environments, are still poorly understood, we know that they contain a selection of polysaccharide-degrading enzymes, and this might be related back to the oil environment in which they were discovered [29].

### Source-driven dynamics of methanogenic microbiomes

Incubating two sources of inocula with the same medium yielded different degradation efficiencies (Fig. 2a, b), as well microbiome dynamics. For example, in the OFPW incubations (Fig. 7a), the O&G group evolved into a quantitatively larger and more diverse methanogenic community compared to the MS group. The O&G community showed higher levels of evenness and richness than the MS community on day 42 (Table 1), which agreed with the fact that the COD removal was 70% greater in the O&G group than in the MS group within 14 days, and on average 40% greater in the O&G group than in the MS group over 42 days (Fig. 2b). It appears that the origin of the O&G sludge, from a pre-adapted environment, is probably the reason why it performs better than the mixed sludge.

### The impact of pollutants and nutrients on microbiome dynamics

We observed an obvious succession in both MS and O&A microbial communities that were incubated with PAHs (Figs. 1, 3), and OFPW (Figs. 7, 8), and previous studies have also revealed the different impacts of pollutants on the same microbiome. For example, Chang et al. [32] observed a clear change in the dominant populations when incubated in naphthalene, and it was different from that of the phenanthrene cultures. The community structure of NA incubations changed marginally (Fig. 4), but much less obviously compared to the other two groups. A more stable community in the NA incubations might be maintained by the addition of easily degradable organics such as acetate and glucose in the BMP solution [33].

Interestingly, the bacterial community was more prone to change than the archaeal one (consisting mainly of methanogens). For example, the relative abundance of the Euryarchaeota group increased by 35 and 90% in the MS and O&G communities, respectively, after 14-day incubations with PAHs, while the total abundance of bacteria decreased accordingly (Additional file 1: Figure S1). Although recalcitrant, both PAHs and NAs can be converted into methane by methanogens in cooperation with some bacterial partners [19, 34]. For example, Christensen et al. [35] identified a syntrophic culture that was capable of methanising two PAHs, i.e. naphthalene and 1-methyl naphthalene, with the bacteria oxidising the PAHs, and the archaea converting the hydrogen generated to methane.

In this study, nutrients (in the form of BMP media) were added to the NA incubations to explore the hypothesis that one could increase the degradation of recalcitrants by varying the quantity of nutrients in the culture. Natural biodegradation processes by the indigenous microbial biomass may be accelerated by manipulating factors such as increasing key nutrients. For example, microbial degradation of contaminants can be enhanced by the addition of nutrients to activate the indigenous microbiome [11]. The effect of nutrients on the degradation of crude oil has been investigated, and it shows that the dosage of a slow-release fertilizer can stimulate the indigenous microbial biomass in oil-contaminated beach sediments [36]. Although the effect of adding nutrients was not obvious in the NAs degradation (data not shown), the dynamics of the microbiome were indeed less active in our NA incubations compared to the other two groups where nutrients were not present. A further study on the long-term effect of nutrients in preserving key microbiomes and their functions in degradation is needed, and would add to the understanding of how to enhance refractory degradation.

### A natural principle guides a smart bioaugmentation

In general, microorganisms can degrade a wide variety of organic contaminants and can adapt to inhospitable environments [37]. Such adaptation is rooted in the theory of environmental stress [38] where a selective stress pushes the microbial strains with the requisite catabolic genes to be activated, enhanced, and enriched in incubations with specialised nutrient sources [39, 40]. Microbial strains derived from a population that are temporally and spatially prevalent in a specific habitat are more likely to persist as an inoculum when re-introduced into a contaminated environment than the one that is alien to such a habitat [41, 42]. Also, the selection of these strains should be carried out on the basis of understanding which microorganisms are common in that contaminated habitat in order to degrade a particular type of recalcitrant contaminant [43]. For the above reasons, an acclimation process allows microorganisms to survive and remain active under unfavourable conditions, and the lack of it could lead to bioaugmentation failures [44].

## Conclusions

In this study, we aimed to understand the systematic and taxonomical structure of the microbial consortia taking part in biodegrading PAHs, NAs, and OFPW, and more importantly, to learn how these communities evolved under different substrate pressures. Two exotic sources of anaerobic/methanogenic biomass were incubated in replicate microcosms that were fed with PAHs, NAs, and OFPW, respectively. The results showed that PAHs, NAs, and OFPW can all rapidly shape the methanogenic microbiomes from exotic sources in as quickly as 14 days. The dominant phyla vary depending on the biomass source, feed type, and variation in nutrients. The methanogens *Methanoregula, Methanosarcina,* and *Methanobacterium* clearly play important roles in the syntrophic degradation of PAHs, NAs, and OFPW, along with saccharolytic fermenters such as *Kosmotoga, Clostridium, Syntrophobacter*. In general, the sludge that originated from an oil and gas processing wastewater treatment (O&G) reactor adapted faster than the one from municipal solid treatment reactors, almost certainly because the O&G biomass had been “pre-selected” by the environment.

## Methods

### Microcosms

The inocula for the microcosms were taken from two mesophilic (35–37 °C) full-scale anaerobic digestion (AD) reactors: one treating oil and gas processing wastewater in Norway (named as O&G sludge), while the other treated mixed biodegradable fractions of municipal solid wastes in the UK (named as mixed sludge). Both the O&G and mixed sludge were filtered through a 500-μm mesh before inoculation to ensure sample homogeneity. The filtered sludge was then incubated in 40-mL serum bottles with a medium that contained solutions for a standard BMP test [33] plus either OFPW, NAs or PAHs.

The synthetic PAH solution was a mixture of five different compounds, including Phenanthrene, Naphthalene, Fluorene, Anthracene, and Fluoranthene, at a concentration of 10 mg PAH mixture per litre. The NA solution contained commercial NA products that were derived from different fractions during petroleum refining at two concentrations of 150 and 500 mg L^−1^. The artificial OFPW contained a salt matrix and crude oil suspension which was prepared using two non-ionic surfactants, Tween 20 and Span 80, to keep the oil as an emulsion, and the final composition of OFPW was similar to a previous study [15].

Each experiment in the test contained a control group (in duplicate), and an assay (in triplicate). For each 40-mL serum bottle, the liquid volume was 36 mL, leaving 10% of the volume as headspace for biogas accumulation. The PAH and OFPW incubations were carried out at fixed concentrations, while the NA incubations were conducted at two levels of concentration, and with different BMP media contents from 50, 100, 200, to 400% (percentage compared to the media composition given by [33]). The final biomass concentration in all the serum bottles was 2.0 g-VSS L^−1^. All the microcosms were incubated for 42 days, but after every 14 days, 10 mL of the completely mixed biomass was removed from each microcosm and anaerobically sub-cultured into a new 40-mL serum bottle that contained the same volume and concentration of media as the mother incubation.

### Molecular biological analysis

At the end of each incubation, 1.8 mL of mixed biomass was sampled from each sub-culture and centrifuged at 15,000×*g* for 10 min; the pellets were then used for DNA extraction. An UltraClean^®^ Microbial DNA Isolation Kit (MoBio Laboratories, CA, USA) was used to extract the DNA following the manufacturer’s instructions. The isolated DNA was stored at −20 °C until qPCR analysis and Illumina MiSeq sequencing by The Genomic Analysis Centre (TGAC, Norwich, the UK). In total over 5,000,000 reads were retrieved from the MiSeq sequencing.

The post-sequencing analysis was done using open-source software QIIME [45] and R [46]. Both *α*- and *β*-diversity indices were analysed based on the sequencing data. The *α*-diversity is defined as the diversity of organisms in one sample or environment, including the following indexes: the observed number of operational taxonomic units (OTUs), Chao1, Shannon index, and InvSimpson, which have been commonly used to characterise microbial diversity in AD reactors [21]. The *β*-diversity is defined as the difference in the microbial diversities across different samples or environments. Commonly used algorithms such as weighted/unweighted UniFrac and Bray-Curtis distances were used to reveal biologically meaningful patterns in the microbiome dynamics.

The qPCR test was performed using an ABI StepOnePlus™ real-time PCR system (Foster City, CA, USA) with two primer sets: Bac516-F-Bac805-R for all bacteria and ARC787-F-ARC1059-R for all archaea. Triplicate PCR reactions were carried out on all samples and negative controls. The thermal cycling program consisted of 2 min at 50 °C, 1 min at 95 °C, followed by 40 cycles of 10 s at 95 °C, 35 s at 56 °C (for Bac516-F/Bac805-R) or 61 °C (for ARC787-F/ARC1059-R). Finally, a melting curve analysis was performed to verify the specificity of the PCR products. The standard curves for the above primer sets were constructed according to previously described methods [20, 47].

## Additional file

**Additional file 1: Figure S1.** The relative abundance at the Phylum level of the communities in the PAH incubations. **Figure S2.** The relative abundance at the Phylum level of the communities in the NA incubations. **Figure S3.** The relative abundance on the Phylum level of the communities in the PW incubations.

## Abbreviations

PAHs: polycyclic aromatic hydrocarbons
NAs: naphthenic acids
OFPW: oil field produced water
qPCR: quantitative polymerase chain reaction
OSN: observed species number
