# Supplementary material for: Dynamics of two methanogenic microbiomes incubated in polycyclic aromatic hydrocarbons, naphthenic acids, and oil field produced water
Source: Biotechnol Biofuels. 2017 May 11;10:123. doi: 10.1186/s13068-017-0812-2 (PMC5426053; doi:10.1186/s13068-017-0812-2)
Supplement: Supplementary file 1 — Additional file 1: Figure S1. The relative abundance at the Phylum level of the communities in the PAH incubations. Figure S2. The relative abundance at the Phylum level of the communities in the NA incubations. Figure S3. The relative abundance on the Phylum level of the communities in the PW incubations. [file 13068_2017_812_MOESM1_ESM.docx]

# Supplementary Information

# Dynamics of two methanogenic microbiomes incubated in polycyclic aromatic hydrocarbons, naphthenic acids, and oil field produced water

Bonahis J. Oko, Yu Tao, David C. Stuckey*

Department of Chemical Engineering, Imperial College London, South Kensington Campus, SW7 2AZ London, the UK

*Corresponding author: David C. Stuckey

E-mail: [d.stuckey@imperial.ac.uk](mailto:d.stuckey@imperial.ac.uk);

Tel: +44 207 5945591

**Fig. S1**

**Fig. S2**

**Fig. S3**
